# Chromosome Painting Based on Bulked Oligonucleotides in Cotton

**DOI:** 10.3389/fpls.2020.00802

**Published:** 2020-06-30

**Authors:** Yuling Liu, Xiuyuan Wang, Yangyang Wei, Zhen Liu, Quanwei Lu, Fang Liu, Tao Zhang, Renhai Peng

**Affiliations:** ^1^Anyang Institute of Technology, Anyang, China; ^2^State Key Laboratory of Cotton Biology/Institute of Cotton Research of Chinese Academy of Agricultural Sciences, Anyang, China; ^3^Jiangsu Key Laboratory of Crop Genetics and Physiology/Key Laboratory of Plant Functional Genomics of the Ministry of Education/Jiangsu Key Laboratory of Crop Genomics and Molecular Breeding, Agricultural College of Yangzhou University, Yangzhou, China

**Keywords:** *Gossypium raimondii*, oligonucleotide, fluorescence *in situ* hybridization, chromosome painting, 45S rDNA

## Abstract

Chromosome painting is one of the key technologies in cytogenetic research, which can accurately identify chromosomes or chromosome regions. Oligonucleotide (oligo) probes designed based on genome sequences have both flexibility and specificity, which would be ideal probes for fluorescence *in situ* hybridization (FISH) analysis of genome structure. In this study, the bulked oligos of the two arms of chromosome seven of cotton were developed based on the genome sequence of *Gossypium raimondii* (DD, 2*n* = 2× = 26), and each arm contains 12,544 oligos. Chromosome seven was easily identified in both D genome and AD genome cotton species using the bulked chromosome-specific painting probes. Together with 45S ribosomal DNA (rDNA) probe, the chromosome-specific painting probe was also successfully used to correct the chromosomal localization of 45S rDNA in *G. raimondii*. The study reveals that bulked oligos specific to a chromosome is a useful tool for chromosome painting in cotton.

## Introduction

Identification of an individual chromosome is the foundation of cytogenetic research. In the early studies, chromosomes can be identified majorly by chromosome morphological characteristics (McClintock, 1929) and chromosome banding techniques (Comings, 1978). Since 1980s, the development of fluorescence *in situ* hybridization (FISH) has led to the development of different chromosome identification techniques owing to its higher sensitivity and resolution. Among them, chromosome painting is one of FISH techniques using chromosome-specific probes to detect specific chromosome regions or an entire chromosome (Pinkel et al., 1988). At early stage, chromosome painting using DNA probes prepared from flow-sorted or micro-dissected chromosomes was successfully used for chromosome identification, chromosome structure analysis, and comparative genomic analysis in human and other mammals with relatively small genomes (Breneman et al., 1993; Scherthan et al., 1994; Rens et al., 2006; Ferguson-Smith and Trifonov, 2007). However, the same type of probes could not work effectively in higher plants with large, complex genomes owing to the poor specificity of probes, which resulted from higher content of repetitive sequences (Fuchs et al., 1996; Lysak and Mandáková, 2013).

In the middle of 1990s, construction of bacterial artificial chromosome (BAC) system in plants enhanced the application of BAC-based FISH in plant cytogenetic research (Woo et al., 1994). Based on pooled BAC clones derived from a specific chromosome, the chromosome-specific painting technique was developed in certain plant species (Lysak et al., 2003, 2010; Ziolkowski et al., 2006; Mandáková and Lysak, 2008). This facilitated the studies on genome structure and evolution. However, this technique also shows some limitations in plant species with complex genome enriched in high repetitive sequences, which cannot guarantee the specificity of the selected BAC contigs.

Sequencing of a large amount of plant genomes and the development of massive parallel synthesis techniques make oligonucleotide (oligo)-based chromosome painting possible in plants. Han et al. (2015) developed a bioinformatic pipeline to select oligos specific to an individual chromosome of cucumber and first successfully applied this technique in plants. Following the application of oligo-based painting in cucumber, this technique has been quickly modified and applied in other plant species for chromosome identification and study on karyotyping, chromosome variations and evolution, such as in strawberry (Qu et al., 2017), *Saccharum spontaneum* (Meng et al., 2018), *Solanum* (He et al., 2018), rice (Hou et al., 2018; Liu et al., 2019), and maize (Albert et al., 2019). To further improve the efficiency, the technology was optimized, and a multiplex PCR-based oligo-painting strategy (MP-OP) was developed, which allows different double-strand-labeled probes to generate from the same oligo library with fluorophore-conjugated nested specific primers (Bi et al., 2019).

The cotton genus (*Gossypium*) is divided into eight monophyletic groups (A–G, and K) (2*n* = 2× = 26), and one allopolyploid lineage (AD) (2*n* = 4× = 52) (Wendel and Albert, 1992; Percival et al., 1999). Up to now, approximately 48 diploids and seven tetrapolyploid cotton species have been identified (Wang et al., 2018). It is believed that A and D diploids diverged 4–8 million years ago and re-hybridized about 1,2 million years ago to form allopolyploid AD lineage (Cronn et al., 2002). Two diploid species, *Gossypium arboreum* and *Gossypium herbaceum*, contain the A genome, whereas the genome D is present in 14 species (Wang et al., 2018). However, the donors of A and D genomes are still unclear. Based on the genetic and morphological evidence, some earlier studies suggested that the allopolyploid cotton is of monophyletic origin (Hutchinson et al., 1947; Wendel and Cronn, 2003), but some others took the point of polyphyletic origin (Johnson, 1975; Parks et al., 1975). Among D genome cotton species, *Gossypium raimondii* has been considered as the most possible paternal donor to the allopolyploids, although other species have been suggested (Wendel et al., 2010). The application of FISH technology using genome-DNA (gDNA) and specific repetitive sequence probes had provided strong evidences for the origin and evolution of the cotton genus (Liu et al., 2005; Xiao et al., 2017; Lu et al., 2018). As a widely used repetitive sequence, 45S ribosomal RNA gene (rDNA), is composed of 18S, 5.8S, and 28S rRNA encoding sequences connected by two internal transcribed spacers (ITS1 and ITS2) (Wendel et al., 1995). Highly repeated 45S rDNA units are arranged in tandem at one or several chromosomal loci (Chang et al., 2010). In *G. raimondii*, the 45S rDNA was located at the terminal region of chromosomes D_5_02, D_5_09, and D_5_11 (corresponding to Chr.05, Chr.06, and Chr.07 of *G. raimondii* genome sequence map), which differ greatly with other D genome species (Gan et al., 2013). The sequence of ITS region (accession number U12718) amplified from *G. raimondii* (Wendel et al., 1995) had been used to blast against the *G. raimondii* genome sequence^1^. Result showed that the *G. raimondii* ITS was mapped on the 38,109,269–38,109,944 bp of chromosome seven with only one hit match, which is inconsistent with previous results in copy number and chromosome position (data unpublished). The distribution of 45S rDNA sites is one of the important references for the study of genome reorganization and phylogenetic analysis (Hanson et al., 1996; Wendel and Cronn, 2003; Chacón et al., 2012). Therefore, the accurate chromosome localization of 45S rDNA is crucial. In this study, we developed oligo pools of *G. raimondii* chromosome seven on the basis of the released whole genomic sequence for chromosome identification as well as 45S rDNA location verification, which would provide evidence for the study of the evolutionary relationship between cotton species.

## Materials and Methods

### Plant Materials

Eleven cotton species were used in this study: four wild species of D genome cotton, *Gossypium thurberi* D_1_, *Gossypium davidsonii* D_3–d_, *Gossypium klotzschianum* D_3–k_, and *G. raimondii* D_5_ (DD, 2*n* = 2× = 26); two A genome cotton, *Gossypium herbacium* A_1_ and *Gossypium arboretum* A_2_ (AA, 2*n* = 2× = 26); and five AD genome cotton, *Gossypium hirsutum* AD_1_, *Gossypium barbadense* AD_2_, *Gossypium tomentosum* AD_3_, *Gossypium mustelinum* AD_4_, *Gossypium darwinii*, and AD_5_ (AADD, 4*n* = 4× = 52). All materials were grown in National Wild Cotton Nursery in Sanya, China. Root tips were harvested from the about 6-day seedlings planted in an incubator and pretreated by 25-ppm cycloheximide at 20°C for 80 min, then fixed in methanol–acetic acid (3:1), and stored at 4°C for 24 h. Metaphase chromosome preparations were prepared according to a previously reported method (Liu et al., 2016).

### Bioinformatics Pipeline for Oligo Selection

Chorus2 software^2^ was used to design the two oligo sets, and it was carried out following published procedure (Albert et al., 2019). Briefly, the *G. raimondii* genome sequence (Paterson et al., 2012)^1^ was divided into 45-nt oligos in step size of 5 nt, and short sequences were mapped to genome, and oligos mapped in two or more locations (with 75% of homology) were eliminated. Then, all respective sequences related oligos were filtered out from oligo set using ChorusNGSfilter.py and ChorusNGSselect.py in Chorus2 package.

### Preparation of Oligo Probes and 45S Ribosomal DNA Probe

Two oligo libraries were synthesized by Synbio Technologies (Suzhou, China). The oligo probes were prepared following the previous protocol (Liu et al., 2019). In brief, through four stages, that is, library amplification with high-fidelity enzyme (KAPA HiFi HotStart ReadyMix, KAPA Biosystems) and T7 *in vitro* transcription with MEGAshortscript^TM^ T7 kit (Invitrogen), reverse transcription using biotin- and digoxigenin-labeled RT primers (5′ dye/CGTGGTCGCGTCTCA), and enzymatic RNA removal. Then, the biotin-labeled or digoxigenin-labeled single-stranded oligo probes were directly used as FISH probes. The probe 45S rDNA was isolated using Plasmid Miniprep Kit (Biomiga) according to the handbook and were labeled with Biotin-Nick Translation Mix (Roche) according to the instructions of the manufacturer^3^.

### Fluorescence *in situ* Hybridization

FISH analysis of single-stranded oligo probes was performed according to a previous protocol (Han et al., 2015). Biotin- and digoxigenin-labeled probes were detected using streptavidin, Alexa Fluor^TM^ 488 conjugate (Invitrogen) and anti-digoxigenin Rhodamine Fab fragments (Roche), respectively. Chromosomes were counter-stained with 4′−6-diamidino-2-phenylindole (DAPI) in Vectashield antifading solution (Vector Laboratories) under a cover slip. Slides were examined under a Zeiss Imager M1 microscope. Images were captured and merged using MetaSystems Isis software with a CCD camera (MetaSystems CoolCube 1) attached to a Zeiss Imager M1 microscope. After the first-round FISH, slides were immersed in 1× phosphate-buffered saline (PBS) to remove the coverslips and were dehydrated in ethanol series (70, 90, and 100%, each for 5 min). Then the repeated FISH was performed according to a previous procedure (Cheng et al., 2001).

## Results

### Development of the Chromosome-Specific Oligonucleotide Sets in *G. raimondii*

**FIGURE 1:**
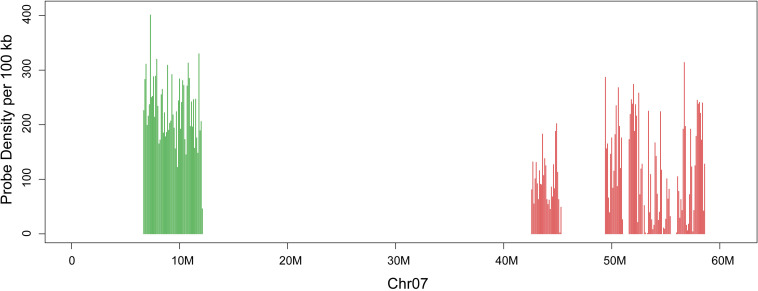
Distribution of two oligo sets on chromosome seven of *Gossypium raimondii*. The *x*-axis shows DNA sequence position on chromosome. The *y*-axis shows the number of oligos in 100-kb windows. Set 1 is in green, and set 2 is in red.

By using the developed bioinformatics pipeline (Albert et al., 2019), two sets of oligos were developed for the two arms of chromosome seven in *G. raimondii* on the basis of the genome assembly. The distribution of the two sets of oligos on chromosome 7 of *G. raimondii* genome sequence map is shown (Figure 1). Set 1 oligo pool (green) consists of 12,544 45-nt oligos spanning 6,710,744–12,163,021 bp of the *G. raimondii* chromosome 7, with the density of 2.3 oligos per kilobase. Set two oligo pool (red) consists of 12,544 45-nt oligos spanning 42,643,131–58,639,905 bp of the *G. raimondii* chromosome seven, with the density of 0.78 oligo per kilobase. To facilitate oligo sequence amplification, every 45-nt oligo contained consistent 5′-forward (T7 RNA polymerase promoter sequence) and 3′-reverse primers.

### Physical Localization of Chromosome 7 Oligo Probes

**FIGURE 2:**
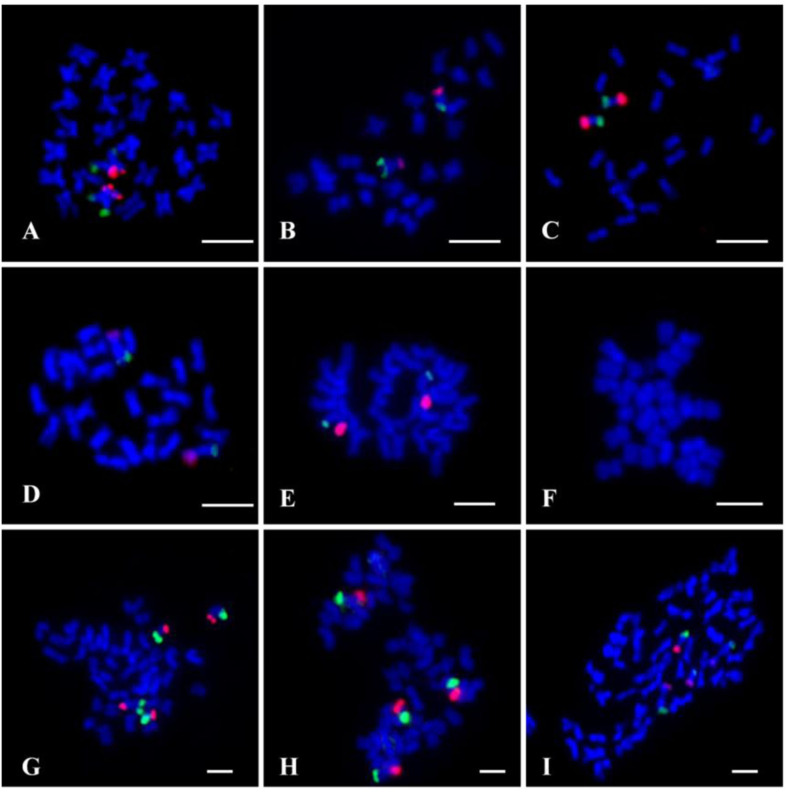
Fluorescence *in situ* hybridization (FISH) using the chromosome 7 oligo probes (set 1, green; set 2, red) on metaphase chromosomes of cotton. **(A)** *Gossypium raimondii* (D_5_). **(B)** *Gossypium thurberi* (D_1_). **(C)** *Gossypium davidsonii* (D_3–d_). **(D)** *Gossypium klotzschianum* (D_3–k_). **(E)** *Gossypium herbacium* (A_1_). **(F)** *Gossypium arboretum* (A_2_). **(G)** *Gossypium hirsutum* (AD_1_). **(H)** *Gossypium barbadense* (AD_2_). **(I)** *Gossypium mustelinum* (AD_4_). Chromosomes were stained with 4′,6-diamidino-2-phenylindole (DAPI). Bars, 5 μm.

To test the sensitivity and reliability of the chromosome 7 oligo probes, set 1 (biotin-labeled) and set 2 (digoxigenin-labeled) oligo probes were hybridized to metaphase chromosomes of *G. raimondii* using double-color FISH. Both probes produced bright FISH signals on one arm of a pair of *G. raimondii* metaphase chromosomes, respectively. The FISH signal from set 2 oligo probe (red) had higher chromosome coverage than that of set 1 oligo probe (green). Additionally, owing to the lower resolution of metaphase FISH, no signal gap was viewed on the corresponding chromosomal region (Figure 2A).

To further verify if the chromosome 7-specific probes selected based on D genome of *G. raimondii* would work for other cotton species, both the two sets of oligo probes were also hybridized to metaphase chromosomes of D genome species (*Gossypium thurberi* D_1_, *G. davidsonii* D_3–d_, and *G. klotzschianum* D_3–k_), *G. herbacium* (A_1_) and *Gossypium arboretum* (A_2_) from A genome, and three AD genome cotton species (*G. hirsutum* AD_1_, *G. barbadense* AD_2_, and *G. mustelinum* AD_4_). The two oligo probes produced signals at the ends of two arms of a pair of D-genome chromosomes, with stronger signals in *G. davidsonii* than *G. thurberi* and *G. klotzschianum* (Figures 2B–D). Probes produced bright FISH signals on a pair of chromosomes of *G. herbacium* (Figure 2E). There were two pairs of chromosome 7 oligo signals in the three tetraploid cotton species (Figures 2G–I), indicating that the oligo probes had signals in both subgenome of tetraploid cotton. However, there was no signal on chromosome of *G. arboretum* (Figure 2F).

### Co-location With the Chromosome 7-Specific Oligo and 45S Ribosomal DNA Probes

**FIGURE 3:**
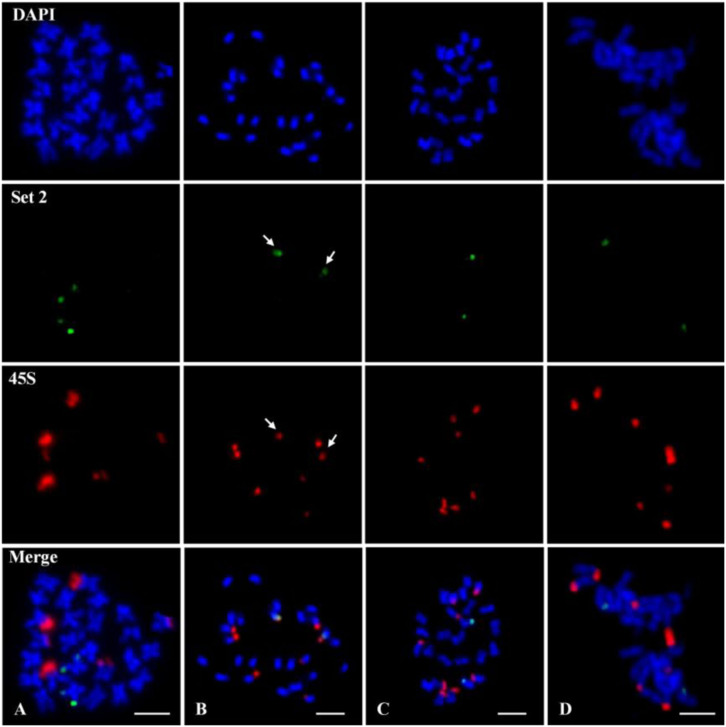
Fluorescence *in situ* hybridization (FISH) using the chromosome 7 set 2 oligo (green) probe and 45S ribosomal DNA (rDNA) (red) probe on metaphase chromosomes of four D genome cotton species. **(A)** *Gossypium raimondii*. **(B)** *Gossypium thurberi*. **(C)** *Gossypium davidsonii*. **(D)** *Gossypium klotzschianum*. The arrows in **(B)** show overlapped signals between 45S rDNA and set 2 oligo. Chromosomes were stained with 4′,6-diamidino-2-phenylindole (DAPI). Bars, 5 μm.

The chromosome 7 of *G. raimondii* genome sequence is homologous to the chromosome 11 (here named as D11) of tetraploid cotton; the former was based on a genetic map (Rong et al., 2004; Paterson et al., 2012), and the latter is based on the physical chromosomes (Wang et al., 2006). A 45S rDNA signal is typically located at the telomeric region on D11 in *G. raimondii* on the basis of the study of Gan et al. (2013). Theoretically, the chromosome 7-specific oligo developed from *G. raimondii* genome sequence should be collinear with one of 45S rDNA locus. To verify this, the chromosome 7-specific oligo probe and 45S rDNA probe were hybridized to the same chromosome preparation of *G. raimondii* (Figure 2A) by repeated FISH. The results indicated that three pairs of 45S rDNA signals (red) were detected on *G. raimondii* chromosomes, but none of them was collinear with the chromosome 7-specific oligo signals (green) (Figure 3A). Next, the chromosome 7-specific set 2 oligo probe and 45S rDNA probe were tested on other three D genome species. Four pairs of 45S rDNA signals (red) were detected on *G. thurberi* chromosomes, and a pair of 45S rDNA overlapped with chromosome 7 set 2 oligo (Figure 3B shown with arrows), which is consistent with a previous study in which there was a 45S rDNA locus on *G. thurberi* chromosome D11 (Gan et al., 2011). FISH results on *G. davidsonii* and *G. klotzschianum* showed no collinearity between chromosome 7-specific oligo and 45S rDNA (Figures 3C,D). This confirmed a previous study in which there were no 45S rDNA loci on chromosomes D11 of these two species (Gan et al., 2012).

**FIGURE 4:**
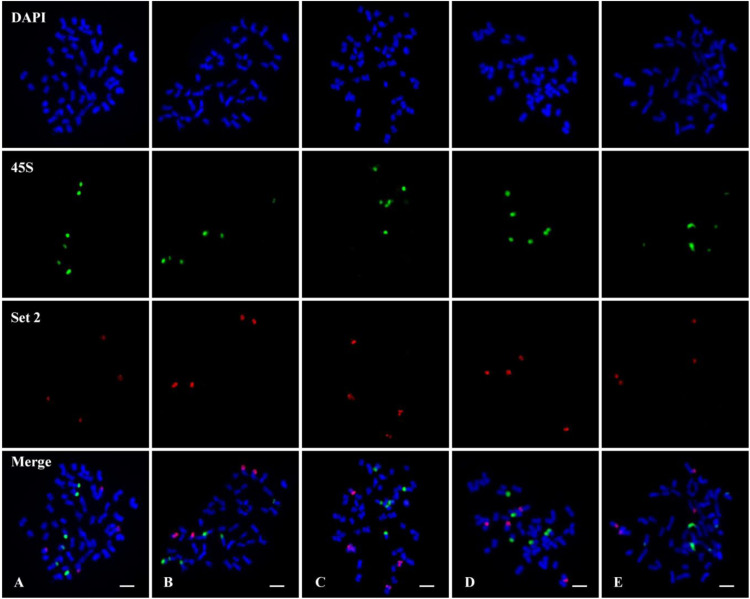
Fluorescence *in situ* hybridization (FISH) using the chromosome 7 set 2 oligo (red) probe and 45S ribosomal DNA (rDNA) (green) probe on metaphase chromosomes of five tetraploid cotton species. **(A)** *Gossypium hirsutum* (AD_1_). **(B)** *Gossypium barbadense* (AD_2_). **(C)** *Gossypium tomentosum* (AD_3_). **(D)** *Gossypium mustelinum* (AD_4_). **(E)** *Gossypium darwinii* (AD_5_). Chromosomes were stained with 4′,6-diamidino-2-phenylindole (DAPI). Bars, 5 μm.

FISH using the chromosome 7 set 2 oligo (red) probe and 45S rDNA (green) probe was performed on metaphase chromosomes of five tetraploid cotton species. All five species showed clear two pairs of set 2 oligo signals on A_t_11 and D_t_11 homoeologous chromosomes (Figure 4, Set 2); each species had three pairs of 45S rDNA signals (Figure 4, 45S), but none of them located are on the same chromosome (Figure 4, Merge).

## Discussion

### The Bulked Oligo Probes Showed High Specificity for Chromosome Identification

FISH technique has become one of the most important techniques in plant molecular cytogenetics owing to its superiority of direct mapping of DNA sequences on cytological specimens (Jiang, 2019). During the application of FISH technology, significant efforts have been made to develop suitable probes to identify chromosomes of different cotton species, such as specific genome repetitive sequence probe for identification of specific chromosome region (Hanson et al., 1996; Liu et al., 2016), genomic DNA probe for genome-specific chromosome painting in polyploid species (Wu et al., 2013), and large-insert genomic DNA clone probe for individual chromosomes identification (Wang et al., 2006; Gan et al., 2011). However, the above-mentioned probes showed different limitations in the application process (Jiang and Gill, 2006). In particular, owing to the more than 60% repetitive sequences in cotton genome (Paterson et al., 2012; Hu et al., 2019), the FISH result of large-insert genomic DNA clone probe may be greatly affected by repetitive sequences, which may show low chromosome specificity. The bulked oligo probes designed based on specific chromosome sequence had demonstrated great superiority in resolution and versatility (Han et al., 2015; Qu et al., 2017; He et al., 2018; Meng et al., 2018). In this study, we developed two oligo probes of *G. raimondii* chromosome 7. Owing to the removal of repetitive sequences from the oligo sets, the two oligo probes produced clear signals without background noise on the chromosome 7 when hybridized to chromosomes of *G. raimondii* and its relative species (Figures 2A–D). At the same time, high proportions of repetitive sequences in the pericentromeric heterochromatic region of chromosomes (Liu et al., 2016; Lu et al., 2019) caused the disappearance of oligos in the middle region of the chromosome (Figure 1), which led to the signals of the oligo probes not diffusing the whole chromosome arms, and only appeared at the two end regions of chromosome (Figure 2). Moreover, the homoeologous chromosomes of *G. raimondii* chromosome 7 in allotetraploid cotton were unambiguously identified using the designed oligo probes (Figures 2G–I). Thus, bulked oligo probes are superior in flexibility, specificity, and repeatability than are the BAC-based probes, which will be useful for studying cotton karyotype evolution.

### No 45S Ribosomal DNA Locates on Chromosome 7 (D_5_11) of *G. raimondii*

rRNA gene is highly a conserved repetitive sequence in the plant genome. Variation in the number and distribution of 45S rDNA signals can indicate genome reorganization and phylogenetic context (Hasterok et al., 2006, 2019; Wolny and Hasterok, 2009; Chacón et al., 2012). As an attractive model for studying the origin, evolution of polyploids, great efforts have been made using 45S rDNA as a probe to study the evolutional relationship of cotton (Hanson et al., 1996; Ji et al., 1999). In *G. raimondii*, the 45S rDNA was located at the terminal region of chromosomes D_5_02, D_5_09, and D_5_11 (corresponding to Chr.05, Chr.06, and Chr.07 of *G. raimondii* genome sequence map) (Gan et al., 2013). That is, there is a 45S rDNA locus at the chromosome 7 of *G. raimondii* according to the chromosome number in the genome sequence. As a component of 45S rDNA, ITS sequence is highly repetitive in the nuclear genome and has become an important molecular marker in the study of phylogeny reconstruction in plant (Wendel et al., 1995). The blast result of ITS region of *G. raimondii* against the *G. raimondii* genome sequence is inconsistent with previous results in copy number and chromosome position (data unpublished). In this study, the result of co-location with the chromosome 7-specific oligo and 45S rDNA probes showed that no 45S rDNA was located at chromosome 7 (D_5_11) of *G. raimondii* (Figure 3A). This is not consistent with previous studies too (Gan et al., 2013) but is in agreement with ITS blast result. Previous identification of 45S rDNA-bearing chromosomes was by means of BAC-FISH using the chromosome-specific BAC clone derived from *G. hirsutum* (AD_1_) (Gan et al., 2013). As allotetraploid, *G. hirsutum* contains high proportions of repetitive sequences, which would make it difficult for genome-derived BAC clones to produce chromosome-specific FISH signals by BAC-FISH (Jiang and Gill, 2006). Owing to the specificity and repeatability of the chromosome-specific oligo probe, it is most likely that no 45S rDNA locates on chromosome 7 (D_5_11) of *G. raimondii*. This result will prompt us to reposition 45S rDNA loci of *G. raimondii* to investigate the evolutionary relationship of cotton.

### *Gossypium herbacium* Was More Likely the Donor of A Subgenome of Tetraploid Cotton

The specificity and presence or absence of oligo signals between different species can reflect the differences in genetic relationships and genomic sequences (Liu et al., 2019). In present study, two pairs of chromosome 7 oligo signals were viewed in both subgenome of tetraploid cotton (Figures 2G–I). Among two A genome species, there was a pair of bright oligo signals on chromosomes of *Gossypium herbacium* (Figure 2E). However, there was no signal on chromosome of *Gossypium arboretum* (Figure 2F). That is, *G. herbacium* generated similar signals with A subgenome of tetraploid cotton, suggesting that *G. herbaceum* was more likely a closer relative to the A subgenome donor of tetraploid cotton than *G. arboreum*, which supported the views of the predecessors by means of visual evidences (Gerstel, 1953; Wendel et al., 2010).

## Conclusion

Using a massive oligo synthesis strategy, we developed the bulked oligos of the two arms of chromosome 7 of *G. raimondii*. The results showed that the chromosome 7 was easily identified in both D genome and AD genome cotton species using the bulked chromosome-specific painting probes. The study reveals that bulked oligos specific to a chromosome is a useful tool for chromosome painting in cotton. Therefore, the oligo-FISH probes we established will be a powerful tool for studying chromosome variations and evolution in the genus *Gossypium*. Based on the established oligo-FISH system, more oligo probes can be designed to study karyotype structure and evolution in more sequenced cotton genomes.

## Data Availability Statement

All datasets generated for this study are included in the article/supplementary material.

## Author Contributions

RP, YL, and TZ conceived the study and drafted manuscript. YL, XW, and YW conducted the cytogenetic experiments. TZ designed the oligo probes. ZL, QL, and FL participated in the data analysis. All authors read and approved the final manuscript.

## Conflict of Interest

The authors declare that the research was conducted in the absence of any commercial or financial relationships that could be construed as a potential conflict of interest.

## Footnotes

1 https://www.cottongen.org/species/Gossypium_raimondii/jgi_genome_221

2 https://github.com/zhangtaolab/Chorus2

3 www.roche-applied-science.com
